# Economic Burden of Legionnaires’ Disease, United States, 2014

**DOI:** 10.3201/eid2701.191198

**Published:** 2021-01

**Authors:** Madeleine Baker-Goering, Kakoli Roy, Chris Edens, Sarah Collier

**Affiliations:** Centers for Disease Control and Prevention, Atlanta, Georgia, USA

**Keywords:** Legionnaires’ disease, cost of illness, absenteeism, mortality, premature, Legionella, bacteria

## Abstract

Through the use of published estimates of medical costs and new calculations of productivity losses, we estimate the lifetime economic burden of 2014 Legionnaires’ disease cases in the United States at ≈$835 million. This total includes $21 million in productivity losses caused by absenteeism and $412 million in productivity losses caused by premature deaths.

Legionnaires’ disease is a severe form of pneumonia that often results in hospitalization (*1*). The disease is caused by the *Legionella* bacteria, which can grow and spread in poorly maintained manmade water systems and can then be inhaled in aerosolized water droplets. In the United States, reported cases of Legionnaires’ disease have been increasing since 2000, yet evidence suggests that many outbreaks are related to failures in building water system maintenance and might be preventable (*2*). Estimates of costs associated with Legionnaires’ disease could help inform prevention efforts.

A recent estimate of the direct medical costs imposed by domestically acquired Legionnaires’ disease in 2014 included $402 million in costs incurred as a result of hospitalizations and emergency department (ED) visits (*1*). Previous studies have not estimated productivity losses caused by Legionnaires’ disease.

We estimate productivity losses caused by absenteeism and premature deaths. These amounts, when combined with existing estimates of medical costs, provide a more comprehensive estimate of the lifetime economic impact of Legionnaires’ disease in the United States for 2014.

## The Study

The analysis estimates the lifetime cost of illness of 2014 Legionnaires’ disease cases in the United States by including direct medical costs and indirect costs, measured in terms of productivity losses caused by absenteeism and premature deaths. Intangible costs (e.g., pain and suffering) are not included. The estimate draws on secondary data from published, peer-reviewed literature and surveillance data (Table 1). All costs are reported in 2014 US dollars.

**Table 1:** Inputs used to estimate economic burden of Legionnaires’ disease, United States, 2014*

Input	Value (range)	Source
Cases of Legionnaires’ diseases, no.	11,000 (7,430–13,300)	Collier et al. (*1*), Table 1, domestically acquired waterborne cases
Hospitalizations, no.	10,800 (7,280–13,100)	Collier et al. (*1*), Table 2, domestically acquired waterborne cases requiring hospitalization
Workdays lost per hospitalization. no.	10 (8–12)	ABCs 2011–2015 (*3*), length of stay (6 d), plus assumed additional prior to hospitalization and for recovery (4 d), minus weekend (2 d). Range assumed.
Proportion of hospitalized cases occurring among working-age patients, %	60	ABCs 2011–2015 (*3*)
Loss per day because of absenteeism, USD	$363	National Occupational Employment and Wage Estimates, Bureau of Labor Statistics hourly earnings for all occupations, 2014 (*4*)
Deaths, no.	995 (655–1,310)	Collier et al. (*1*), Table 2, fatal, domestically acquired, waterborne cases
Value per premature death (weighed average), USD	$413,727	Grosse et al. (*5*),† updated to 2014 USD by using employment cost index (*6*)

*ABCs, Active Bacterial Core surveillance; USD, 2014 US dollars. †We used the estimates of the present value of lifetime production, which were discounted at 3%.

The estimates (and range) of the incidence, healthcare utilization, and medical costs of Legionnaires’ disease for 2014 were drawn from published sources (*1*). We used estimates of waterborne and domestically acquired cases, hospitalizations, and deaths. We assumed all Legionnaires’ disease deaths occurred in hospitalized patients and estimated the associated costs, which comprise medical expenses and productivity losses caused by premature death. The costs associated with nonfatal hospitalized patients included medical expenses and productivity losses caused by workdays lost because of hospitalization and the subsequent recovery period.

The human capital approach, frequently used in cost-of-illness analysis, was used to value productivity losses (i.e., costs of time lost from productive activities) caused by illness and premature death (*7*). Productivity loss for nonfatal cases requiring hospitalization was estimated as the product of workdays lost per case and the average daily wage rate, among the cases occurring in the working-age population (Table 1). We valued lost workdays by using an average hourly wage rate of $22.71 for 2014 and doubled that rate to account for benefits and overhead (*4*). We assumed that 60% of nonfatal cases requiring hospitalization occurred among working-age persons, on the basis of the age distribution of case-patients from the Centers for Disease Control and Prevention’s Active Bacterial Core surveillance program (ABCs) during 2011–2015 (*3*). We calculated the range by using the 5th and 95th credible interval of cases (*1*) and the low and high range for workdays lost.

Productivity losses caused by premature death were calculated by using age-specific estimates of future lifetime earnings, which included market earnings and the value of household services (*5*). Estimates of earnings were discounted at 3% to account for losses incurred in future years. Earnings were updated to 2014 dollars according to the Bureau of Labor Statistics’ employment cost index (*6*). We calculated the proportion of deaths by age category on the basis of data from ABCs during 2011–2015 (*3*). Deaths with no age reported were valued at the lowest estimates (i.e., equivalent to a death of a person >80 years of age). We calculated the range by using the 5th and 95th credible interval of deaths (*1*).

Table 2 shows the lifetime costs associated with Legionnaires’ disease. An estimated 5,183/8,639 nonfatal cases in hospitalized working-age patients resulted in ≈59,540 workdays lost and $21.3 million in productivity losses. In addition, the 995 premature deaths caused by Legionnaires’ disease resulted in an estimated $412 million in productivity losses. Collier et al. (*1*) estimated approximately $402 million in medical expenses incurred as a result of hospitalizations ($401.0 million) and ED visits ($0.5 million). The estimated total lifetime cost of illness associated with an estimated 11,000 domestically acquired cases of Legionnaires’ disease in 2014 is $835 million (range $362 million–$2.263 billion).

**Table 2:** Economic burden of Legionnaires’ disease, United States, 2014

Type of cost	Estimate (range)
Medical costs (1) for estimate and range	$402 million ($80–$1,690 million)
Productivity losses from workdays lost*	$21,634,454 ($11–$31 million)
Productivity losses from premature deaths	$411,658,786 ($271–$542 million)
Total economic burden	$835,035,255 ($362–$2,263 million)

*Productivity losses from workdays lost were calculated among nonfatal cases requiring hospitalization (10,800 patients – 995 deaths = 9,805 nonfatal cases requiring hospitalization), of which 60% are assumed to be among working-age persons (5,883). Each hospitalized patient is assumed to lose 10 workdays because of hospitalization (5883 × 10 = 58,830 workdays lost); each workday is valued at $363.36.

These estimates depend directly on the rate and age distribution of fatalities. Although half of fatal cases occurred in persons >65 years of age, 74% of the total productivity losses caused by premature deaths were for patients <65 years of age, because future lifetime earnings decrease with age. Also, age was not reported in ABCs for 13% of fatal cases, and productivity losses for estimated fatal cases were valued conservatively (i.e., equivalent to a death occurring in a person >80 years of age). Productivity losses caused by premature deaths might be greater if these deaths occurred among younger persons.

## Conclusions

Productivity losses caused by Legionnaires’ disease account for just more than half of its lifetime economic burden, almost all of which (95%) is caused by productivity losses from premature deaths. The human capital approach used in this analysis provides a conservative estimate of the disease’s indirect costs because it only accounts for productivity losses in its cost estimate of lost workdays and premature deaths. An alternative approach is to estimate the indirect costs of illness and death in terms of societal value or willingness to pay for averting them. This approach would also include such intangible costs as pain and suffering. For illustration, calculated at a constant value of $9.3 million/death, the economic value of the 995 deaths from Legionnaires’ disease cases in 2014 would be $9.3 billion (*8*). This approach is most commonly used in cost-benefit analyses to consider tradeoffs between the societal benefits of a policy, including the value society places on fewer deaths, and the costs of implementing a policy.

Our analysis is subject to several limitations. Key limitations that apply to the case-number estimate (*1*) will also apply here, such as reliance on a series of multipliers, including some generated by structured expert judgment, and lack of information on sensitivity of the urinary antigen test for all *Legionella* species and serogroups. We excluded some costs because of lack of available data. However, because estimates suggest that >90% of persons with Legionnaires’ disease are hospitalized or treated in the ED (*1*), these excluded costs (e.g., outpatient non-ED medical expenses and workdays lost for persons who are not hospitalized) are likely small. In addition, we estimated the economic value of workdays lost for all nonfatal cases occurring among working-age persons; however, whether all persons who contracted Legionnaires’ disease were employed is unknown, so the financial costs incurred might be lower.

In conclusion, our analysis indicates that the economic burden of Legionnaires’ disease more than doubles when lifetime productivity losses are added to medical costs. Our estimate can help demonstrate the value of investments in preventing Legionnaires’ disease, such as water management programs and outbreak investigations.
